# Unraveling the Enigma of Postablation Rhabdomyolysis: Diagnostic Dilemmas and Therapeutic Strategies

**DOI:** 10.7759/cureus.115844

**Published:** 2026-09-06

**Authors:** Alex Fernandez, Nicole Nudelman, Ann K Valencia, Gustavo A Hernandez, Nishka R Jakkidi, Jeffrey Valencia Uribe, Brian G Nudelman

**Affiliations:** 1 Allopathic Medicine, Herbert Wertheim College of Medicine, Florida International University, Miami, USA; 2 Osteopathic Medicine, College of Medicine, Kansas City University, Kansas City, USA; 3 Critical Care Medicine, University of South Florida, Tampa, USA; 4 Internal Medicine, Memorial Healthcare System, Pembroke Pines, USA

**Keywords:** anesthesia complications, atrial fibrillation ablation, creatine phosphokinase, malignant hyperthermia, propofol infusion syndrome, rhabdomyolysis

## Abstract

Rhabdomyolysis is characterized by the breakdown of skeletal muscle tissue and the release of intracellular contents into the circulation, and it poses diagnostic challenges due to its heterogeneous presentation and potential for serious complications. We present a case of a 44-year-old male with a history of hypertension, anxiety, and persistent atrial fibrillation (AF) who developed rhabdomyolysis the morning after a scheduled catheter ablation. He presented with myalgias, muscle cramps, weakness, and brown-colored urine; laboratory studies revealed a creatine phosphokinase (CPK) of 23,757 U/L, hyponatremia, and mildly elevated transaminases despite a normal urinalysis. His CPK improved to below 5,000 U/L after 72 h of intravenous fluid resuscitation, and he was discharged following electrolyte correction.

Differential diagnoses considered included propofol infusion syndrome, position-related (pressure-induced) rhabdomyolysis, metabolic myopathy, and malignant hyperthermia (MH), with MH emerging as the most probable etiology given his exposure to succinylcholine and the close temporal relationship between anesthesia and symptom onset. Definitive confirmation was limited by the patient's decision to decline muscle biopsy and genetic testing. Outpatient electromyography did not show a myopathic process, and the patient later tolerated a cardioversion performed under a non-triggering anesthetic protocol without recurrence. This case underscores the importance of maintaining a high index of suspicion for subclinical or atypical MH and anesthesia-induced rhabdomyolysis (AIR) in patients without a prior history of anesthetic complications, and it highlights the diagnostic complexity that arises when confirmatory testing is unavailable or declined. Further research into the mechanisms underlying subclinical MH, along with standardized diagnostic and monitoring protocols for perioperative rhabdomyolysis, is warranted to optimize patient care in similar scenarios.

## Introduction

Rhabdomyolysis signifies the breakdown of skeletal muscle tissue, resulting in the release of intracellular components such as myoglobin, creatine phosphokinase (CPK), urate, potassium, and phosphate into the bloodstream; it is traditionally characterized by the clinical triad of myalgia, generalized muscle weakness, and tea-colored urine [1]. Rhabdomyolysis stems from various etiologies, broadly categorized into traumatic and non-traumatic causes. Traumatic instances include crush injury and direct trauma, while non-traumatic causes encompass overexertion, seizures, infections, adverse drug reactions, skeletal muscle ischemia, intoxications, malignant hyperthermia, and metabolic syndromes [1,2]. However, the classical triad manifests in only a minority of patients, with many remaining asymptomatic upon initial presentation. Diagnosis predominantly relies on elevated CPK levels, the most sensitive biomarker for muscle injury [3]. Supplementary diagnostic measures aim to detect potential complications, including a comprehensive metabolic panel to assess electrolyte imbalances and renal function, arterial blood gas analysis to evaluate acid-base disequilibrium, and electrocardiography for monitoring cardiac arrhythmias. Complications arising from the pathophysiology of rhabdomyolysis include elevated serum CPK and myoglobin leading to pigment nephropathy and toxin-induced acute tubular necrosis, hyperkalemia or hypocalcemia-triggered cardiac arrhythmias, hypovolemia due to fluid sequestration into damaged muscle tissue, and compartment syndrome from direct muscle injury or reperfusion [4]. The cornerstone of therapy lies in intravenous fluid resuscitation, identification and reversal of underlying causes, correction of electrolyte imbalances, and vigilance for potential complications.

Malignant hyperthermia (MH) is a potentially life-threatening pharmacogenetic disorder of skeletal muscle characterized by an abnormal hypermetabolic response to triggering anesthetic agents, most notably volatile anesthetics and succinylcholine [1,2]. Anesthesia-induced rhabdomyolysis (AIR) refers to acute skeletal muscle breakdown occurring in temporal association with anesthetic exposure and may overlap clinically with MH, making recognition of perioperative anesthetic exposure particularly important when evaluating otherwise unexplained postoperative rhabdomyolysis [1,2]. We present a case of a 44-year-old male with persistent atrial fibrillation (AF) who developed rhabdomyolysis a day after a scheduled electrophysiology study ablation. This case underscores the diagnostic challenges encountered and emphasizes the critical importance of early detection of anesthesia-induced rhabdomyolysis to mitigate future anesthesia-related complications.

## Case presentation

A 44-year-old male with a past medical history of hypertension, anxiety, and persistent AF presented to the emergency department (ED) with myalgias, muscle cramps, and weakness that started the morning after his catheter ablation for AF. He denied any associated shortness of breath, chest pain, palpitations, headache, or fevers. He reported that his pain was also accompanied by brown-colored urine; however, he denied any dysuria or urgency. On physical examination, the patient was alert, oriented, and in no acute distress. Vital signs were notable for a temperature of 98.6°F (37.0°C), blood pressure of 124/76 mmHg, heart rate of 125 beats/min, respiratory rate of 18 breaths/min, and oxygen saturation of 98% on room air. Cardiovascular examination revealed a regular rhythm and tachycardia without murmurs, rubs, or gallops. The lungs were clear to auscultation bilaterally. The abdomen was soft, non-tender, and non-distended, and there was no peripheral edema or focal neurologic deficit, but there were bilateral upper and lower extremity spasms. In the ED, laboratory studies were notable for mild leukocytosis and anemia, severe hyponatremia, mild hypokalemia, hypochloremia, hypocalcemia, and hypomagnesemia, as well as markedly elevated aspartate aminotransferase (AST), mildly elevated alanine aminotransferase (ALT), and elevated troponin. In the absence of chest pain or ischemic changes on EKG, the troponin elevation was attributed to controlled myocardial injury created during AF ablation. Renal function was preserved (Table 1). An electrocardiogram (EKG) showed sinus tachycardia with a rate of 128 beats/min (bpm) (Figure 1). Chest radiography showed no acute cardiopulmonary process (Figure 2). The patient's electrolytes were repleted, and he was admitted for further evaluation. The following morning, additional laboratory testing revealed a markedly elevated creatine phosphokinase (CPK) level of 23,757 U/L (Figure 3). In the setting of his muscle spasms and reported dark urine, empiric treatment for rhabdomyolysis was initiated. Interestingly, urinalysis returned normal without any evidence of blood or myoglobin; however, urine osmolality was low at 120 mOsm/kg. Urine drug screen was negative. Upon further questioning, the patient reported that he generally exercised for 45 min per day and did not partake in any extreme exercise prior to or after his ablation. He also denied any history of seizures. After three days of continuous intravenous (IV) lactated Ringer's, the patient's CPK decreased to below 5,000 U/L (Figure 3). The electrophysiology team was consulted and concluded that the patient had an acute episode of rhabdomyolysis that may have been secondary to subclinical malignant hyperthermia (MH); they recommended outpatient follow-up with neurology for further testing (Table 2). The patient was discharged on hospital day four with instructions to continue his beta-blocker and apixaban and to follow up with both electrophysiology and neurology as an outpatient.

**Table 1:** Patient’s initial laboratory values obtained at presentation. Reference ranges are representative of adult ranges and may vary by laboratory and assay; sex-specific ranges reflect values for an adult male.

Laboratory parameters	Patient value	Reference range
White blood cell count (WBC)	12.2×10³/µL	4.5-11.0×10³/µL
Hemoglobin (Hgb)	11.8 g/dL	13.5-17.5 g/dL (male)
Blood urea nitrogen (BUN)	12.0 mg/dL	7.0-20.0 mg/dL
Creatinine	1.09 mg/dL	0.7-1.3 mg/dL (male)
Sodium	122 mmol/L	135-145 mmol/L
Potassium	3.3 mmol/L	3.5-5.0 mmol/L
Chloride	90.0 mmol/L	96.0-106.0 mmol/L
Calcium, total	8.2 mg/dL	8.5-10.5 mg/dL
Magnesium	1.4 mg/dL	1.7-2.2 mg/dL
Aspartate aminotransferase (AST)	432.0 U/L	8.0-40.0 U/L
Alanine aminotransferase (ALT)	73.0 U/L	7.0-56.0 U/L
Troponin I	1.513 ng/mL	<0.04 ng/mL (assay-dependent)
Creatine phosphokinase (CPK), hospital day 2	23,757 U/L	30.0-200.0 U/L (male)
Creatine phosphokinase (CPK), hospital day 4	<5000.0 U/L	30.0-200.0 U/L (male)
Urine osmolality	120.0 mOsm/kg	300.0-900.0 mOsm/kg (random)

**Figure 1:**
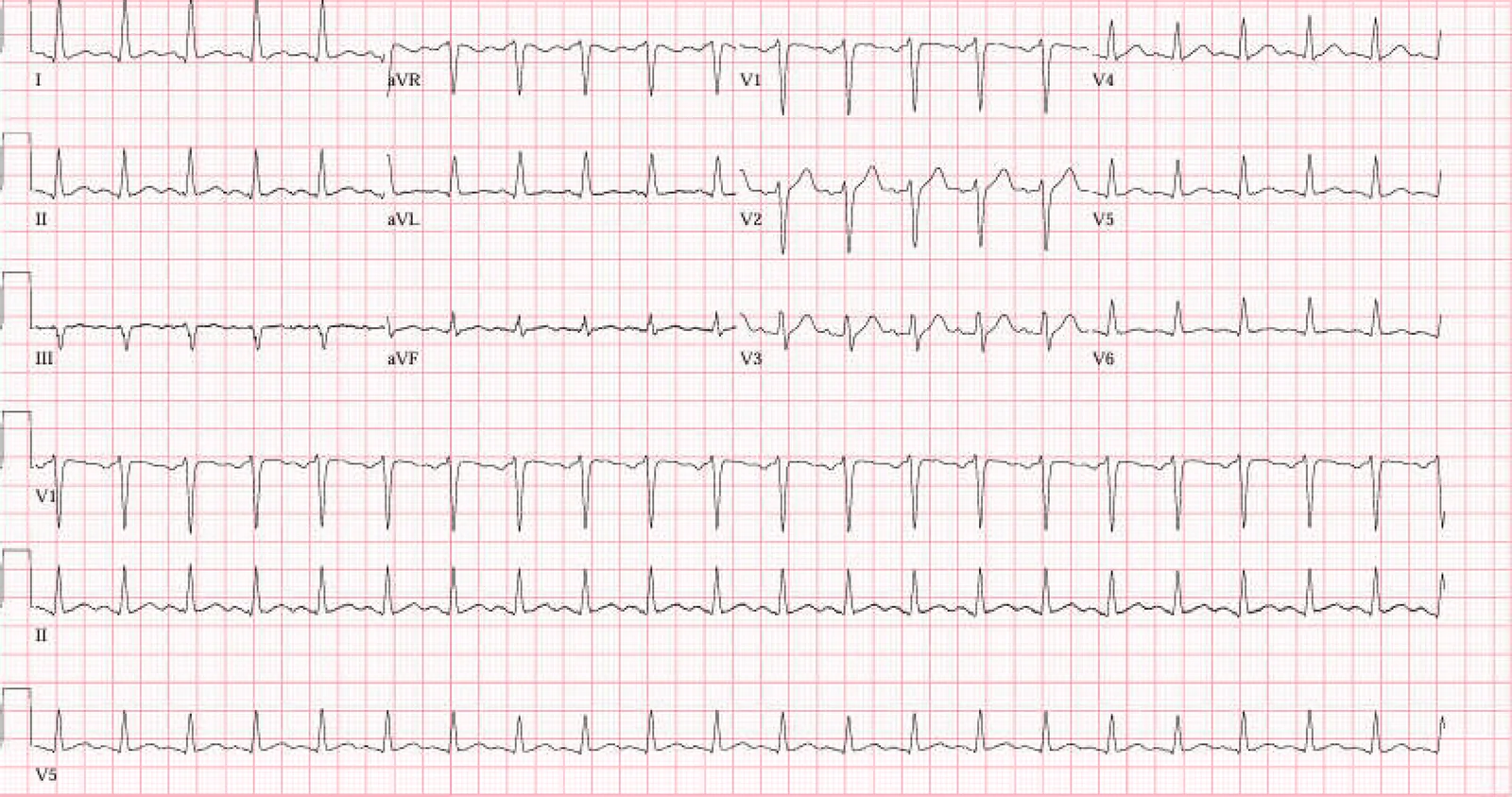
Initial electrocardiogram demonstrating sinus tachycardia at 128 beats/min without ischemic changes or evidence of atrioventricular block.

**Figure 2:**
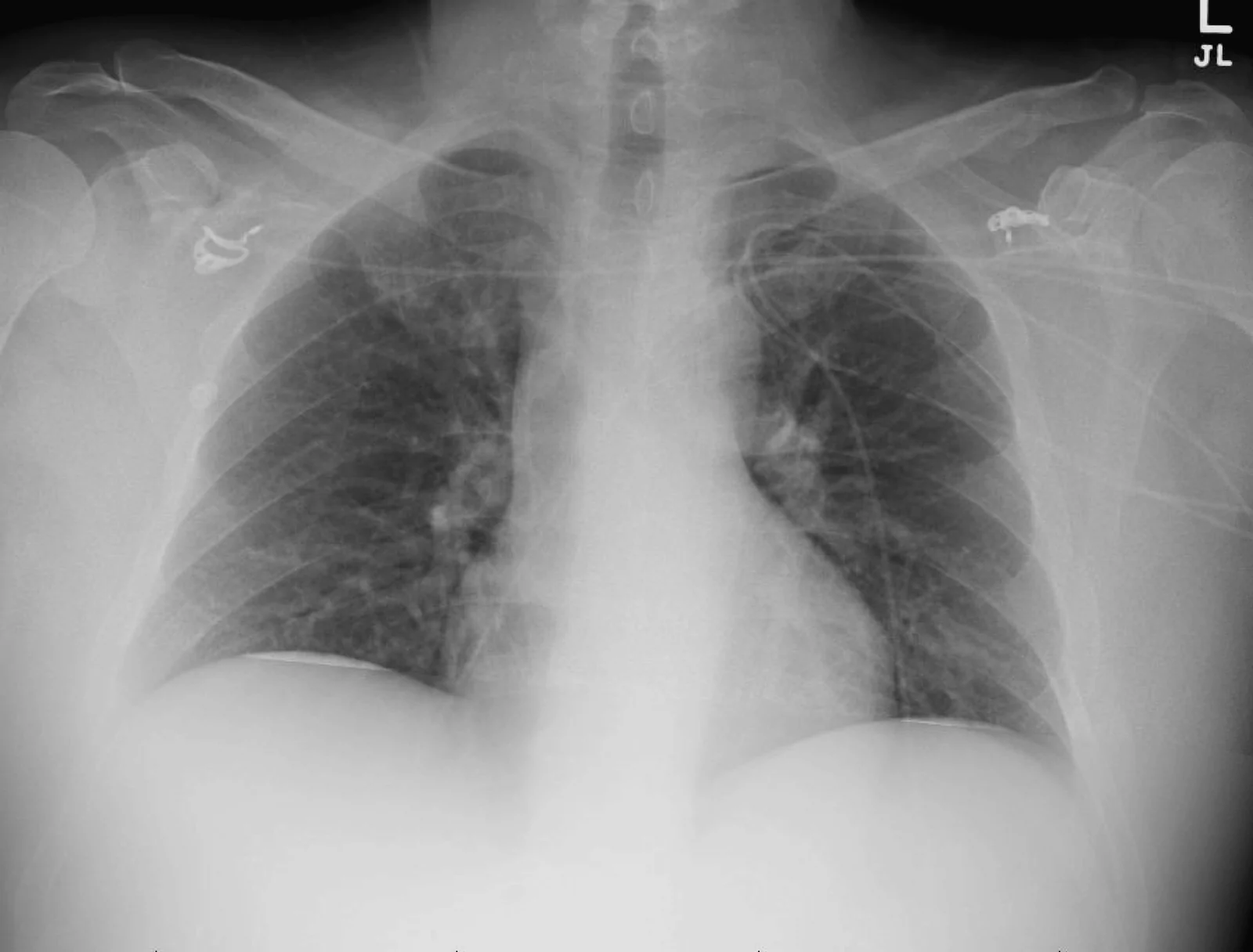
Chest X-ray on admission showing no acute intrapulmonary process with a normal cardiac silhouette and a normal mediastinum.

**Figure 3:**
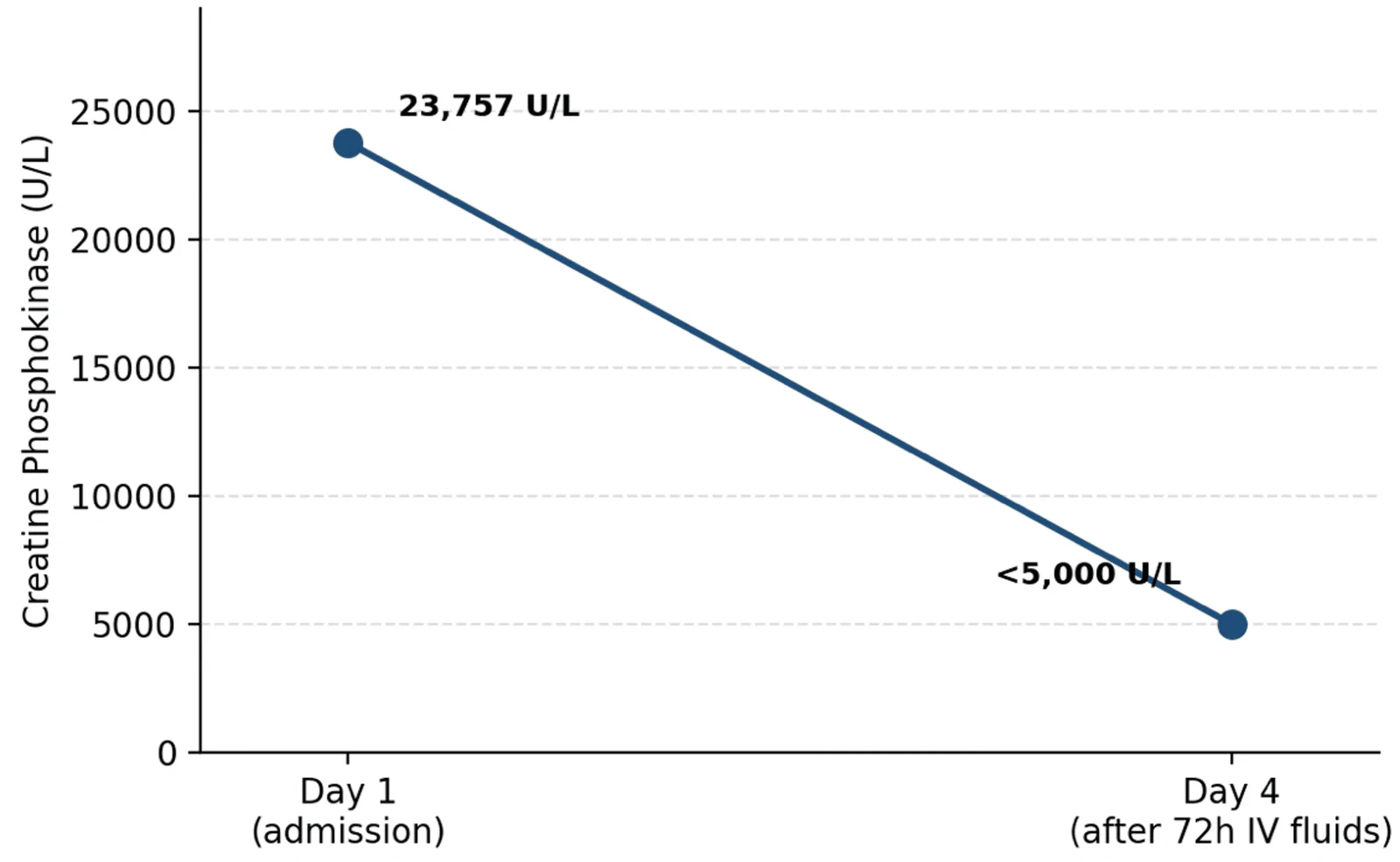
Trend in serum creatine phosphokinase (CPK) during hospitalization. CPK fell from 23,757 U/L to below 5,000 U/L after 72 h of intravenous fluid resuscitation (reference range {male}: 30-200 U/L).

**Table 2:** Differential diagnosis considerations. This table includes some of the most plausible differentials for our patient's presentation, along with the pathophysiology/risk factors for each. CPK: creatine phosphokinase; AKI: acute kidney injury; ATP: adenosine triphosphate

Differential diagnosis	Presentation	Pathophysiology	General risk factors	Factors supporting diagnosis in our patient	Factors against diagnosis in our patient
Propofol infusion syndrome (PRIS)	High anion gap metabolic acidosis, rhabdomyolysis/elevated CPK, bradycardia/arrhythmia, myalgias	Elevated propofol levels disrupt the electron transport chain and fatty acid metabolism, and impair beta-adrenergic/calcium channel receptor binding	Prolonged propofol infusion (>48 h), high doses (>4 mg/kg/h)	Rhabdomyolysis, myalgias	No acidosis (normal anion gap), no bradycardia/arrhythmia, only ~30 mg propofol administered, propofol infused for <1 h
Position-related (pressure-induced) rhabdomyolysis	Rhabdomyolysis/elevated CPK, swelling of large muscle groups/signs of compartment syndrome, myoglobinuria/AKI/acidosis, myalgias	Sustained pressure on large muscle groups during prolonged surgery impairs local perfusion and ATP production and raises cytosolic calcium, precipitating myocyte injury	Extensive bariatric or intrathoracic procedures, obesity, prolonged surgical time, prone/lateral decubitus positioning	Rhabdomyolysis, myalgias	Minimally invasive procedure (<20 min), BMI: 27 kg/m², no muscle swelling or signs of compartment syndrome, supine positioning
Malignant hyperthermia (MH)	High fevers, hypotension, cardiac arrhythmias, acidosis, hyperkalemia, muscle rigidity/myalgias, rhabdomyolysis	Continuous calcium release from the sarcoplasmic reticulum causes uncontrolled muscle contraction, resulting in breakdown of muscle tissue and release of intracellular contents	Inhalational halogenated anesthetics, succinylcholine administration	Succinylcholine administered, no prior history of anesthesia with an offending agent, rhabdomyolysis, myalgias	No fever, no hypotension, no arrhythmia, hypokalemia (rather than hyperkalemia)

A week later, outpatient nerve conduction testing and electromyography (EMG) performed by neurology did not show evidence of a myopathic process. The patient decided not to proceed with caffeine-halothane contracture testing or RYR1 mutation testing. Soon after the initial hospitalization, the patient returned to the hospital with palpitations and weakness and was found to be in atrial flutter. He was started on propafenone three times daily, given one dose of amiodarone, and scheduled for cardioversion without transesophageal echocardiography. The procedure was performed with a specialized, non-triggering anesthetic protocol using propofol, etomidate, and lidocaine as a precaution against another episode of MH, and the patient was successfully converted to sinus rhythm without complication. He was discharged home on propafenone, metoprolol, and apixaban, and continued close outpatient follow-up with electrophysiology. Three months later, propafenone was discontinued given maintenance of normal sinus rhythm (NSR). The patient had no additional episodes of muscle cramping, twitching, or dark urine during this period, and repeat CPK was within normal limits.

## Discussion

Rhabdomyolysis typically manifests with a triad of symptoms as follows: myalgia, weakness, and dark-colored urine or myoglobinuria. However, it is important to note that the presence of all three symptoms is not necessary for diagnosis, as fewer than 10% of cases exhibit this classical triad [1]. Our patient displayed all three symptoms, yet his urinalysis on admission showed no evidence of dark coloration or myoglobin. Despite this, his markedly elevated CPK level of 23,757 U/L strongly supported the diagnosis. Treatment with intravenous fluids, electrolyte replacement, and monitoring is crucial for successful management of this condition [1]. While acute renal failure is the most common life-threatening complication, our patient maintained normal levels of blood urea nitrogen (BUN), creatinine, and urine output throughout his hospital stay [5]. In the present case, risk factors such as hypertension, propofol administration, and succinylcholine use during the procedure may have contributed to the development of rhabdomyolysis [1,5,6]. The most plausible differential diagnoses include propofol infusion syndrome (PRIS), position-related (pressure-induced) rhabdomyolysis, and malignant hyperthermia (MH). Through this discussion, we present evidence supporting each of these diagnoses and explain why MH emerged as the most likely explanation for our patient's presentation.

Beyond these three principal considerations, the differential diagnosis for perioperative rhabdomyolysis should also include inherited metabolic myopathies, such as glycogen storage disorders and fatty acid oxidation defects, which can precipitate anesthesia-induced rhabdomyolysis (AIR) [1,2]. These conditions were considered less likely in our patient given the acute, single-episode nature of his rhabdomyolysis in close temporal relationship to anesthesia exposure, in contrast to the recurrent, exercise-triggered episodes that typically characterize metabolic myopathies and that usually begin earlier in life. Neuromuscular diseases, including the muscular dystrophies (e.g., Duchenne's and Becker's) and congenital myopathies (e.g., central core disease and King-Denborough syndrome), also merit brief mention, as patients with these conditions carry an increased risk of both classic MH and AIR when exposed to volatile anesthetics or succinylcholine [7,8]. Notably, a normal electromyogram (EMG), as obtained in our patient, does not reliably exclude an underlying metabolic myopathy or channelopathy, as interictal EMG and laboratory testing are frequently normal in these disorders, and diagnosis often requires forearm exercise testing, muscle biopsy, or genetic analysis [9]. Given the absence of a personal or family history of exercise intolerance, recurrent myoglobinuria, or fixed weakness, however, these diagnoses were felt to be less likely contributors to our patient's presentation than PRIS, position-related rhabdomyolysis, or MH.

Propofol infusion syndrome (PRIS) is a rare but severe complication of propofol administration that can potentially lead to fatal outcomes. Typically, PRIS occurs following prolonged infusion exceeding 48 h or administration at high doses surpassing 4 mg/kg/h [10]. Elevated levels of propofol in the body disrupt the electron transport chain, interfere with fatty acid metabolism, and impair beta-adrenergic/calcium channel receptor binding, thereby predisposing to cardiac arrhythmias. The clinical presentation of PRIS includes high anion gap metabolic acidosis, rhabdomyolysis, bradycardia, and arrhythmias [10]. Our patient displayed only one of these hallmark features (rhabdomyolysis) and presented without acidosis, with a normal anion gap and tachycardia rather than bradycardia. Furthermore, he received only approximately 30 mg of propofol over less than 1 h, well below the thresholds associated with PRIS. We therefore turned to our next potential diagnosis, i.e., position-related rhabdomyolysis.

Position-related (pressure-induced) rhabdomyolysis, typically observed after extensive surgeries such as bariatric or intrathoracic procedures, occasionally manifests even after less invasive surgeries. For instance, in 2012, a 34-year-old male developed rhabdomyolysis and bilateral lower extremity muscle swelling following a craniotomy for tumor resection [11]. Contributing factors included sedation with propofol and the use of isoflurane; discontinuation of propofol led to significant improvement, with the rhabdomyolysis ultimately attributed to PRIS. Similarly, in 2015, rhabdomyolysis occurred after ablation for AF in a morbidly obese patient, illustrating the heightened risk in obese patients due to mechanical compression of muscle against the operating table [12]. Recommendations to mitigate this risk include increased padding, regular patient repositioning, and limiting procedure duration [12]. In the present case, however, the patient's BMI of 27 kg/m², short procedure duration (<20 min), supine positioning, and lack of visible muscle swelling or signs of compartment syndrome make these factors unlikely contributors to his rhabdomyolysis.

Our final and most probable consideration is malignant hyperthermia (MH), which is generally characterized by high fevers, hypotension, cardiac arrhythmias, acidosis, hyperkalemia, and muscle rigidity [13]. MH typically arises from inhalation of halogenated anesthetics or administration of succinylcholine, leading to continuous calcium release from the sarcoplasmic reticulum that causes uncontrolled muscle contraction, resulting in breakdown of muscle tissue and release of intracellular contents [13]. Our patient's past surgical history included only a minor procedure under conscious sedation with propofol, without any known postoperative complications. During the catheter ablation, he received 20 mg of succinylcholine, 1 mg of midazolam, 10 mg of rocuronium, 150 μg of fentanyl, and approximately 30 mg of propofol. Although the timing of his symptom onset is consistent with a clinical picture of anesthesia-induced rhabdomyolysis, his presentation did not match the typical, fulminant MH picture. Diagnosing MH during an acute event is presumptive and based on the clinical signs discussed above, as well as a rise in end-tidal carbon dioxide despite increasing minute ventilation; however, these findings were not captured or reported intraoperatively in our patient.

A case from 1998 described a similar scenario in which a patient who had previously been sedated with succinylcholine without complication developed MH after receiving succinylcholine during a radical prostatectomy [14]. As with our patient, his only findings were an elevated CPK level and myoglobinuria, without fever, signs of compartment syndrome, or arrhythmias. He was treated with IV fluids, mannitol, and a sodium bicarbonate drip to prevent acute renal failure, and was started on patient-controlled analgesia to manage severe muscle aches [14]. The diagnosis of MH in this patient was established with muscle biopsy using caffeine-halothane contracture testing, and his presentation was concluded to represent a rare, subclinical form of MH incited by anesthesia.

Unfortunately, despite recommendations for genetic testing and muscle biopsy, our patient chose to forgo further investigation and instead opted to avoid triggering anesthetics in the future. Consequently, we cannot definitively confirm an acute case of MH. Additionally, there is no apparent mechanism by which the catheter ablation procedure itself could have directly induced rhabdomyolysis in this case. Although the etiology of subclinical MH remains incompletely understood, it is established that mutations in multiple genes, including RYR1, CACNA1S, and STAC3, can contribute to MH susceptibility, and it is conceivable that different mutations may manifest with varying degrees of clinical severity [13].

Postoperative isolated rhabdomyolysis has been documented in a limited number of cases involving general anesthesia, summarized in Table 3 [14-17]. Distinguishing whether these occurrences stem from classic MH or from alternative genetic predispositions leading to AIR remains uncertain, as MH may initially present with muscular symptoms and rhabdomyolysis, followed by delayed onset of more classic signs such as hyperthermia, tachycardia, tachypnea, and elevated end-tidal carbon dioxide. Among the four literature cases summarized in Table 3, two patients developed rhabdomyolysis several days postoperatively and were managed with fluid resuscitation alone, without dantrolene [14,15], while the remaining two developed rhabdomyolysis within hours of surgery, with dantrolene administered in one case and withheld in the other based on the absence of hemodynamic instability, life-threatening arrhythmias, or uncontrolled hypermetabolism [16,17]. Isolated myoglobinuria as the sole presenting sign of MH has similarly been reported in both the pediatric and adult literature, further supporting the concept that MH can present along a spectrum ranging from fulminant crisis to subclinical, myopathy-limited disease [18-20]. These observations emphasize the complexity of diagnosing and managing postoperative rhabdomyolysis, particularly in the context of potential MH involvement, and highlight the need for vigilant monitoring and individualized therapeutic approaches to optimize patient outcomes in such scenarios.

**Table 3:** Comparative summary of reported cases of perioperative rhabdomyolysis revealing malignant hyperthermia susceptibility. NR: not reported in the available published summary; CHCT: caffeine-halothane contracture testing; IVCT: in vitro contracture test; POD: postoperative day; EMG: electromyography; CPK: creatine phosphokinase; CK: creatine kinase

Studies	Presenting sign(s)	Time to onset	Anesthetic trigger	Dantrolene	MH testing (method/result)
Harwood and Nelson (1998) [14]	Back pain, elevated CK, myoglobinuria	POD 2	NR	No	CHCT; multiple fascicles abnormal, exceeding thresholds → MH susceptible
McKenney and Holman (2002) [15]	Malaise, myalgias, nausea/vomiting, dark urine	Dark urine day of surgery; represented POD 3	NR	No	CHCT (North American protocol); all strips abnormal → MH susceptible
Evans et al. (2002) [16]	Muscle aches (jaw, neck, triceps, back); CK: 100,030 U/L; urine myoglobin: 796,000 mg/L	~4 h postoperatively	NR	Yes	IVCT; all bundles abnormal → MH susceptible
Fierobe et al. (1998) [17]	Severe perioperative rhabdomyolysis	Perioperative	NR	NR	Contracture testing reported to reveal MH susceptibility; further detail not available
Present case	Myalgias, cramps, weakness, brown urine; CPK: 23,757 U/L	Morning after ablation (~POD 1)	Succinylcholine, midazolam, rocuronium, fentanyl, propofol	No	Patient declined biopsy/RYR1 testing; EMG without myopathic process

## Conclusions

In summary, our case illustrates a rare instance of postoperative rhabdomyolysis occurring in a patient undergoing catheter ablation for AF. While the classical triad of symptoms associated with rhabdomyolysis may not always manifest concurrently, maintaining vigilance for atypical presentations is paramount for prompt diagnosis and intervention. Differential diagnoses including propofol infusion syndrome, position-related rhabdomyolysis, and malignant hyperthermia were thoroughly considered, with MH emerging as the most likely scenario based on clinical features. However, due to the patient's decision to forgo further testing, definitive confirmation of MH remained elusive. Nevertheless, our case underscores the importance of considering rare complications during the postoperative period. For patients without a history of tolerating inhaled halogenated anesthetics or succinylcholine, we advocate for inpatient monitoring extending for at least 12-24 h postoperatively. Although there is currently no established therapeutic protocol for subclinical MH, prioritizing treatment directed at the rhabdomyolysis itself is imperative to mitigate potential complications. Further exploration into the mechanisms underlying subclinical MH and its clinical implications is warranted to deepen our understanding and improve patient outcomes in similar scenarios.
